# Full-color structured illumination optical sectioning microscopy

**DOI:** 10.1038/srep14513

**Published:** 2015-09-29

**Authors:** Jia Qian, Ming Lei, Dan Dan, Baoli Yao, Xing Zhou, Yanlong Yang, Shaohui Yan, Junwei Min, Xianghua Yu

**Affiliations:** 1 State Key Laboratory of Transient Optics and Photonics, Xi’an Institute of Optics and Precision Mechanics, Chinese Academy of Sciences, Xi’an 710119, China

## Abstract

In merits of super-resolved resolution and fast speed of three-dimensional (3D) optical sectioning capability, structured illumination microscopy (SIM) has found variety of applications in biomedical imaging. So far, most SIM systems use monochrome CCD or CMOS cameras to acquire images and discard the natural color information of the specimens. Although multicolor integration scheme are employed, multiple excitation sources and detectors are required and the spectral information is limited to a few of wavelengths. Here, we report a new method for full-color SIM with a color digital camera. A data processing algorithm based on HSV (Hue, Saturation, and Value) color space is proposed, in which the recorded color raw images are processed in the Hue, Saturation, Value color channels, and then reconstructed to a 3D image with full color. We demonstrated some 3D optical sectioning results on samples such as mixed pollen grains, insects, micro-chips and the surface of coins. The presented technique is applicable to some circumstance where color information plays crucial roles, such as in materials science and surface morphology.

Over the past few decades, varieties of optical sectioning technologies have been invented and played increasingly important roles in the researches of biomedical sciences. The confocal microscopy and the two-photon microscopy are the most regularly used techniques for obtaining high quality of optically sectioned images^1^,^2^,^3^. Fundamentally, both of them involve raster scanning point source of excitation laser and detecting the fluorescence signal with photomultiplier tube detectors. With the emergence of new fluorescent molecular probes that can bind proteins with high specificity, the multiple labeling allows the visualization of multiple protein interactions in living cells simultaneously^4^. Besides, multiple labeling also provides improved imaging contrast and definition. High-end multicolor scanning microscopes developed so far are based on the multi-channel integration geometry. Multiple laser excitation sources and detectors for different color channels are employed, and the signals from each channel (red, green, and blue) are detected sequentially and combined into a single file^5^,^6^,^7^. Laser scanning microscopies have axially sectioning capability and high spatial resolution, but the high power of laser may be harmful to living tissues. Besides, non-optical-sectioning methods for full-color 3D imaging, such as spectroscopic optical coherence tomography based on low-coherence interferometry^8^,^9^, have also been developed. However, their spatial resolutions are limited to a few of microns.

Wide-field fluorescence microscopies with optically sectioning power, including light-sheet microscopy^10^,^11^,^12^ and structured illumination microscopy (SIM)^13^,^14^,^15^,^16^,^17^ have recently received lots of attentions due to the advantages of high spatial resolution, short image recording time, and less photobleaching. SIM has found numerous applications for time-lapse imaging of living tissues and cellular structures. It was first invented by Neil *et al.*^13^ as a method of eliminating the out-of-focus background encountered in the wide-field microscopy. It has been demonstrated that the axial resolution of SIM is equal to that of laser scanning microscopy^17^. Furthermore, Gustafsson *et al.*^18^,^19^,^20^,^21^ exploited the SIM to improve the spatial resolution of microscopy, i.e., super-resolution SIM. In this paper, we only focus on the optically sectioning SIM. By projecting a sinusoidal fringe pattern onto the specimen, SIM images the fringe efficiently only on the parts of the specimen that are in focus. The out-of-focus background can be removed by decoding the in-focus information. The most commonly used decoding algorithm was proposed by Neil *et al.*^13^ For each slice, three raw images with an adjacent phase-shift of 2π/3 are obtained. By taking the root mean square (RMS) of the differences of the each two adjacent images, an optically sectioned image can be reconstructed. Recently, Mertz *et al.*^22^,^23^ also introduced a new algorithm called HiLo imaging to synthesize an optically sectioned image, which uses two wide-field images acquired under structured and uniform illumination, respectively. The optically sectioned image is reconstructed by combining the in-focus high and low frequency components.

So far, most SIM systems use monochrome CCD or CMOS cameras to acquire images and discard the natural color information of the specimens. Here we propose a method for full-color SIM system by using a color CMOS camera. The color digital camera can produce color image is due to the use of Bayer filter. The Bayer filter is a special filter array for arranging trichromatic color filter elements on the pixels of the image sensor. The three trichromatic channels are not independent with each other and there must be a spectrum overlap between them. Because each single pixel is filtered to respond to only one of the three colors, the signal from each pixel cannot determine the trichromatic values by itself. To acquire an image with full-color, color restore algorithms developed by the camera manufacturers are used to calculate a set of complete trichromatic values for each pixel, which use the neighbor pixels of the relevant colors to identify the values for a specific pixel. For the multicolor microscopes based on the multi-channel integration geometry, there is a narrow band-pass filter put in front of each detector, the integrated image cannot restore the full-color information of the specimen. So, the use of color digital camera could provide a much broader color range compared with the multi-channel integration geometry.

The CCD or CMOS cameras used in majority of the wide-field microscopes have a pixel depth of 12 or 16 bits that are able to obtain 4096 or 65536 gray scales. In most instances, this is sufficient for processing the typical amount of photoelectrons that a single pixel can contain. However, because the optically sectioning decoding algorithm executes a subtraction operation (see Eq. (1)), this will cause a reduction in the gray scales of images^24^, which makes the color restoration of multi-channel integration scheme distorted. Image processing methods can stretch the existing pixel values to fill the full dynamic range, but cannot add any new information. As a result, when three post-processing images from R, G, B channels with only 1000 out of a possible 4096 gray scales are stretched to fill the full dynamic range for example, the resulting integrated color image appears color cast artifact.

To solve the above issue for a full-color structured illumination optical sectioning microscopy, we propose a new scheme based on our developed Digital Micro-mirror Device (DMD)-based LED-illumination SIM system^16^, in which a color CMOS camera and a white-light LED illumination is employed to realize SIM with full natural color. In contrast to other SIM techniques, the DMD-based LED-illumination SIM is cost-effective, ease of multi-wavelength switchable and speckle-noise-free. A new data processing algorithm based on the HSV (Hue, Saturation, Value) color space is developed, which transforms the recorded three raw images in RGB color space into the HSV color space, and calculates the slicing images in the three HSV channels separately, and then recombines to a 3D image with full-color. To our knowledge, it is the first time to realize 3D SIM image for both fluorescence and reflection imaging with full natural color.

## Results and Discussion

**Figure 1:**
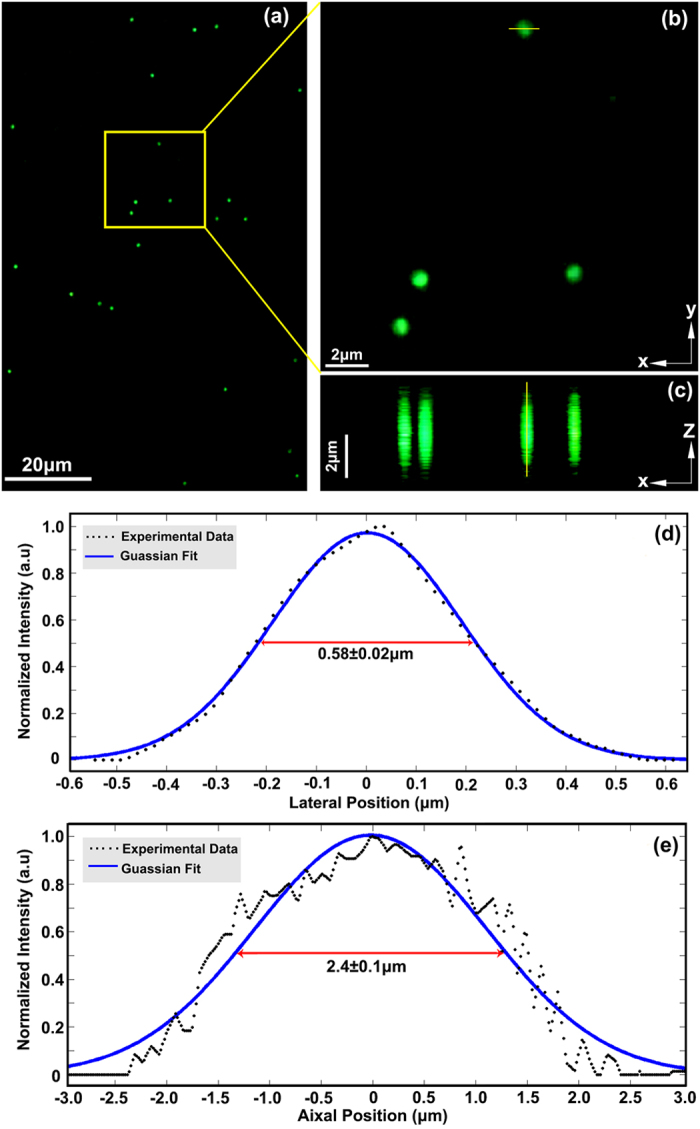
Evaluation of the spatial resolutions using 170 nm green fluorescent microspheres. (**a**) Maximum-intensity projection of 170 nm fluorescent microspheres. (**b**) Zoom-in lateral view (XY-plane) of the rectangular box in (**a**). (**c**) Reconstruction of XZ-plane section of the fluorescent microspheres by an axial slice step of 0.05 μm. (**d**) Line-scan profile of a selected fluorescent microsphere along the cut-line in (**b**). (**e**) Line-scan profile of a selected fluorescent microsphere along the cut-line in (**c**). The experiment was performed with the 20 × /NA0.45 objective at excitation wavelength 475 nm and fluorescent emission wavelength 520 nm.

In order to evaluate the spatial resolution of the color SIM microscope, we used 170 nm in diameter green fluorescent microspheres (520 nm emission@475 nm excitation) as test samples. The size of the microsphere is far below the resolution limit of the microscope using the objective of 20×/NA0.45. The intensity distributions of the 170 nm fluorescent beads in lateral and axial planes are shown in Fig. 1d,e. We sliced 225 layers at the axial stepping interval of 50 nm and captured 675 raw images, so the optical sectioning depth is 11.2 μm. The Gaussian fits and statistical values of the full width at half maximum (FWHM) over 50 microspheres indicate that the lateral and axial resolutions of the system are 0.58 ± 0.02 μm and 2.4 ± 0.1 μm, respectively, which are close to the theoretical resolution limits.

**Figure 2:**
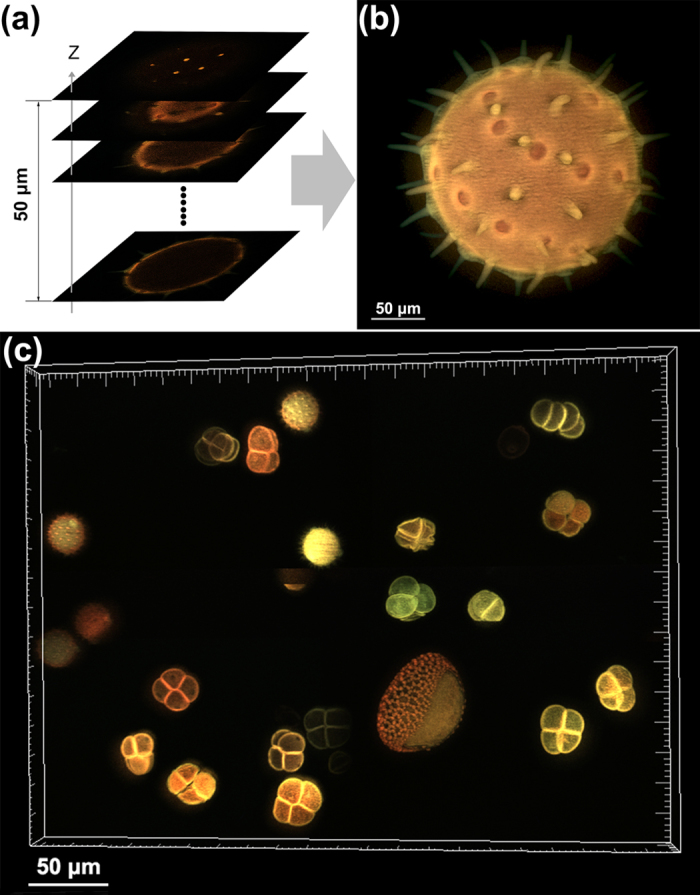
Optical sectioning images of the thick volume structure of different pollen grains. The auto-fluorescence signals of the pollen grains are excited by 405 nm light and collected by 20 × objective. (**a**) A sequence of optically sectioned images. (**b**) The maximum-intensity projection of all the planes along Z-axis. (**c**) The maximum-intensity projection of differently shaped and colorful pollen grains (Supplementary Movie 1).

An experimental result of a mixed pollen grain specimen that emits strong auto-fluorescence signals under the illumination of 405 nm light is shown in Fig. 2. A dichroic mirror (420 nm long-pass, 45^o^ incident angle) is used to separate the illumination and the fluorescence signals. Another long-pass filter (420 nm long-pass, 0° incident angle) is set before the color camera to block the additional illumination light. The long-pass filters set guarantee a broad range of the auto-fluorescence signals in the visible range can be detected by the color camera. Figure 2a presents a sequence of 251 layers (1280 × 1024 pixels/layer) of a volume with 50 μm in depth and sliced with the axial interval of 200 nm. The total data acquisition time is 164 seconds, that is, (215 ms exposure time + 0.031 ms DMD switching time) × 3 patterns × 251 layers + 10 ms Z-stage settling time × 250 axial slice intervals. The maximum-intensity projection image of the 251 layers along Z-axis is shown in Fig. 2b. The data acquisition speed for the color SIM system is restricted by the sensitivity and speed of the color CMOS camera. At the present system, a color CMOS camera with 10 bits gray depth and 60 fps full-frame rate is adopted. The acquisition speed can be much higher by using more sensitive and faster cameras, for instance, using a scientific color digital camera with a filter array of cyan-magenta-yellow combination that has higher transmittance efficiency.

It is known that different pollen grains have different shapes and sizes. Actually, they also emit different colors of auto-fluorescence under the same excitation. To demonstrate this feature, we used our setup to image the mixed pollen grain specimen in different positions. After that, we put these 3D sectioning images together in a single volume to exhibit the colorful pollen grains. Figure 2c presents the maximum-intensity projection of 135 planes under the excitation of 405 nm light by stitching four positions in the specimen. The differently shaped and colorful pollen grains are clearly revealed. The Supplementary Movie 1 gives the “3D color image” of the mixed pollen grains after 3D reconstruction viewed from different angles.

**Figure 3:**
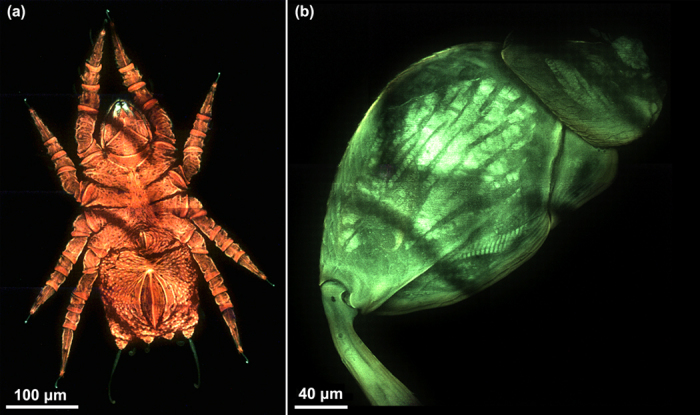
Results of maximum-intensity projection observed with 20 × objective. (**a**) A Congo red stained mite under 405 nm LED light excitation (Supplementary Movie 2). (**b**) A Clavicornaltica’s hindleg femora under 450 nm LED light excitation (Supplementary Movie 3).

The results of maximum-intensity projection of a Congo red stained mite and a Clavicornaltica’s hindleg femora are shown in Fig. 3a,b, respectively. For the mite, we sliced 105 layers at 800 nm axial intervals and captured 315 raw images for each data set, excited by the wavelength of 405 nm. The whole image is stitched by 8 data sets, and the field-of-view (FOV) is 0.407 × 0.610 mm^2^. It is recognized that the mouth and the tentacles of the mite emit differently fluorescent colors distinguished from the body. The Clavicornaltica’s hindleg femora, excited by the wavelength of 450 nm, emits auto-fluorescence signal. Here a 475 nm long-pass dichroic mirror and filter are used to separate the excitation and fluorescence signals. We sliced 223 layers at 800 nm axial intervals and captured 669 raw images for each data set. The whole image is stitched by 4 data sets, and the FOV is 0.410 × 0.324 mm^2^. The Supplementary Movie 2 and Supplementary Movie 3 present the “3D color images” of the mite and the Clavicornaltica’s hindleg femora after 3D reconstruction viewed from different angles.

**Figure 4:**
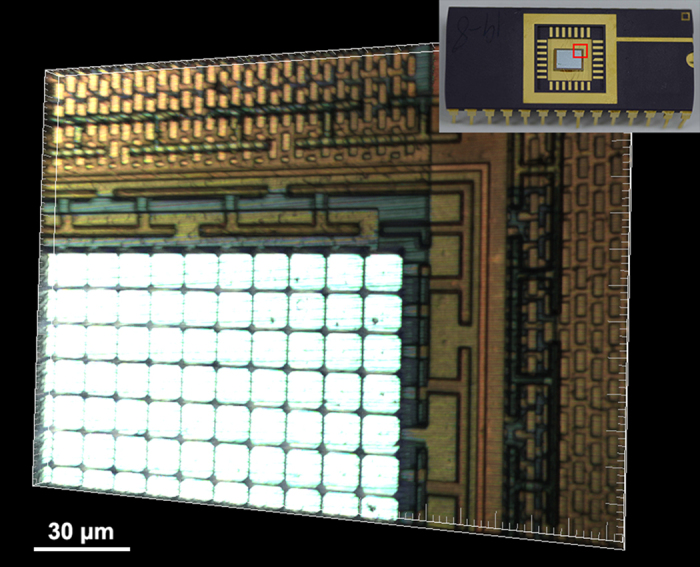
Reconstructed 3D color image of a piece of DMD micro-chip. The signal comes from the reflection of the metallic surface illuminated by a white-light LED and collected by 20 × objective (Supplementary Movie 4).

Since the DMD-based color SIM system is in epi-detection geometry, it is also suitable for mapping of surface morphology. For example, we used the system to acquire the 3D structure of the metallic surface that has strong reflection to the visible light. Here a white-light LED is applied to illuminate the metallic sample. In order to collect the reflective light from the surface, a 50/50 beam-splitter is used instead of the dichroic mirror, and the long-pass filter before the camera is also removed. Before experiment, a calibration of white balance for the color CMOS camera is adopted by using a silver-coated mirror as a sample. Figure 4 shows a 3D color image of a piece of DMD micro-chip rendered from the optically sectioned 25 planes along Z-axis with the 20 × objective, where different colors correspond to different materials. The data acquisition time for the 25 slicing layers is 2.3 seconds, that is, (27 ms CMOS exposure time + 0.031 ms DMD switching time) × 3 patterns × 25 layers + 10 ms Z-stage settling time × 24 axial slice intervals. Because of the strong reflective signal from the sample, the exposure time of camera is set much shorter than that for the mixed pollen grains, thus, the data acquisition time is greatly reduced. The Supplementary Movie 4 shows the “3D color image” of the micro-chip after 3D reconstruction viewed from different angles.

**Figure 5:**
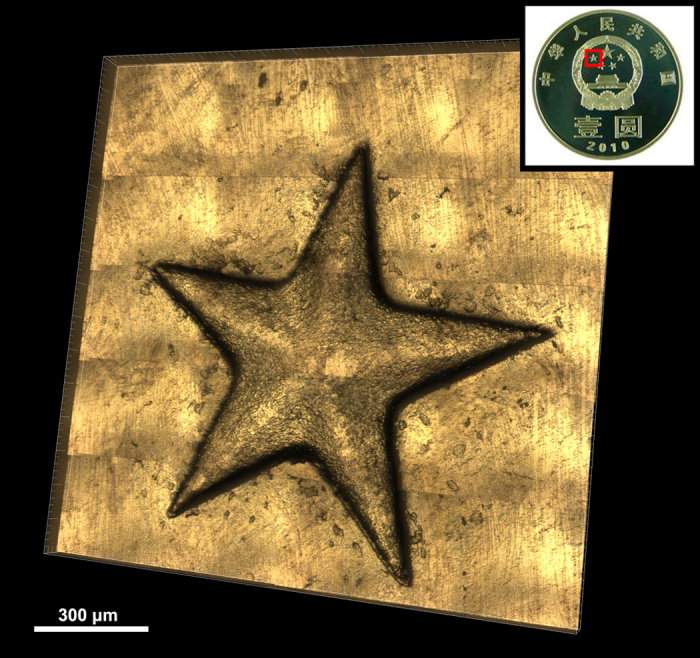
Reconstructed 3D color image of a convex star on a Chinese commemorative coin observed with 10 × objective. The 3D volume is rendered from 20 data sets stitching. The whole FOV is 1.505 × 1.528 mm^2^ (Supplementary Movie 5).

For 3D morphology of objects, techniques such as surface profiling and phase imaging are well known^25^,^26^,^27^. Both of the techniques depend on the phase-shifting scheme. The phase information of an object can be retrieved according to a phase unwrapping algorithm that recovers the true optical path difference map of the specimen. But for sudden and abrupt phase changing objects, such as specimens with high roughness surface or large step height, this method will fail^28^. Recently, Nguyen *et al.*^29^ proposed a method based on multi-view image fusion scheme for capture of natural-color 3D models of insects. It used the color texture extracted from the specimen to map the 3D model of the reconstructed object. But this method is not available for getting structural details, such as steps or hairs. Differing from the above methods, SIM can measure objects with sudden and abrupt phase changing with high resolution and high SNR, moreover, with the natural color restoration of the specimen. It is also possible to get the surface morphology of large specimen whose dimension is beyond the FOV of the objective lens. To do this, we implemented the image stitching technique of multiple images array captured by scanning the sample on the motorized XY stage. Distinctive features are extracted from adjacent FOVs and then matched. The exact overlap among sub-images and identical exposures are crucial to avoiding conspicuous object cut and color inconsistency^30^. This technique provides a submicron resolution and a large FOV up to 2.25 mm^2^ in the experiment. As an example, we took the surface morphology of a coin with the 10 × objective, whose FOV is 0.457 × 0.365 mm^2^. Figure 5 presents a stitched 3D color image of a convex star on a Chinese commemorative coin. The whole FOV is 1.505 × 1.528 mm^2^ by stitching 20 data sets. For each data set, 173 layers of the volume at Z-step of 500 nm are captured. So, 10380 raw color images in 1280 × 1024 pixels are obtained, corresponding to a total data capacity of about 38 Gbytes. The Supplementary Movie 5 shows the “3D color image” of the convex star after 3D reconstruction viewed from different angles.

In summary, we have proposed a scheme for full-color structured illumination microscopy (C-SIM) based on DMD fringe projection and LED illumination, which has practical value to acquiring the broadband fluorescence signals and natural color of reflected light from object surfaces in three dimensions, rather than the artificial color that usually generated from gray-scale data post-processing. With the built apparatus, we are able to obtain optical sectioning images with full natural color of fluorescent specimens or reflection-type objects. We have demonstrated some 3D optical sectioning results on samples such as mixed pollen grains, insects, micro-chips and metallic surface of coins. This technique may find potential applications in such fields as biology (e.g. the study of structural color mechanism of animal^31^), materials science, microelectronics, where color information may play crucial roles.

## Methods

### Color SIM system

**Figure 6:**
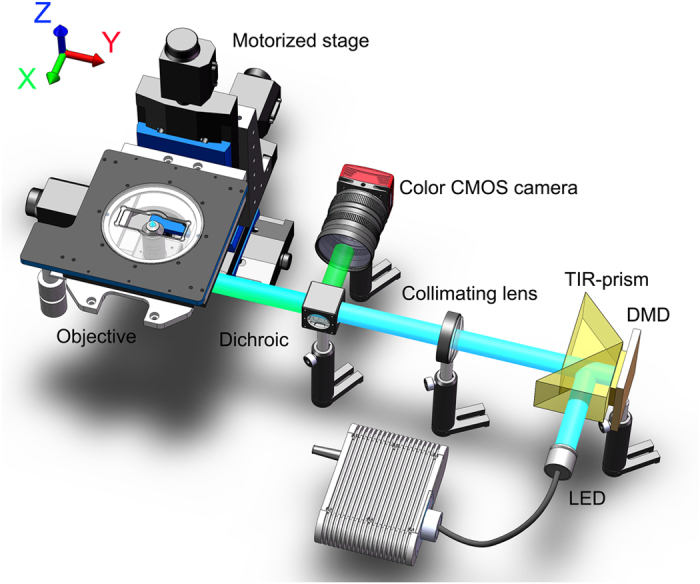
Schematic diagram of the color SIM.

Figure 6 illustrates the schematic diagram of the proposed color SIM apparatus. Four-wavelength (405 nm, 450 nm, 475 nm and white light) high-power LEDs are applied as the illumination sources. A total internal reflection (TIR) prism is used to separate the projection and the illumination paths. The LED light is reflected by the TIR-prism and illuminates the DMD chip. The modulated light then passes through achromatic collimating lens, dichroic mirror, and focused by the objective lens (either a 10 × objective, NA = 0.45, Edmund Optics Inc., USA or a 20 × objective, NA = 0.45, Nikon Inc., Japan) to illuminate the sample. The sample is mounted on a XYZ motorized translation stage (3-M405, Physik Instrumente Inc., Germany) with a minimum movement of 50 nm. For each axial plane in Z-scanning, three fringe-illuminated raw images with an adjacent phase-shift of 2π/3 are captured. Volume data for different axial layers are obtained by axial moving the specimen at different Z positions to acquire the three-dimensional light intensity distribution of the specimen. Moving the specimen stage in XY directions under the objective enable to extend the FOV by use of the image stitching technique. A USB3.0 color CMOS camera with a maximum full-frame rate of 60 fps (DCC3240C, 1280 × 1024 pixels, 10 bits gray depth, Thorlabs Inc., USA) is used to record the 2D images. DMD controlling, image collection, stage movement and image stitching are carried out by custom developed software programmed in C + + .

### Optically sectioning decoding algorithms

Structured illumination is introduced to wide-field fluorescence microscopy as a method of getting higher axial resolution and discriminating against out-of-focus background. A sectioned image is obtained from the RMS of the sum of squared differences between 3 raw images of the specimen given by Eq. (1), with each raw image captured under sinusoidal fringe illumination by a phase shift of 2π/3 mutually^13^.


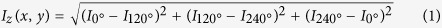


Simultaneously, the conventional wide-field image can also be obtained with


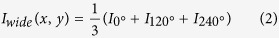


This implies that the optically sectioned image and the wide-field image of the sample can be obtained from the same raw data.

It is known that the gradation of color of an image is dependent on the gray scales of the digital camera. It is always expected to fill the available pixel depth in the data acquisition procedure. Since the scanning microscope measures a single pixel each time, which means that the microscope spends only 0.2 microseconds measuring the fluorescence signal for every pixel at a speed of 5 frames per second for an image with 1000 × 1000 pixels for example. Whereas, for CCD/CMOS array detectors a two-dimensional image is captured for all pixels in parallel, which means much longer per-pixel measurement time is allowed at even higher frame rate. Then more photons can be collected and more dynamic range of such images can be obtained. For these reasons, wide-field images are particularly well suited for digital image processing approaches.

**Figure 7:**
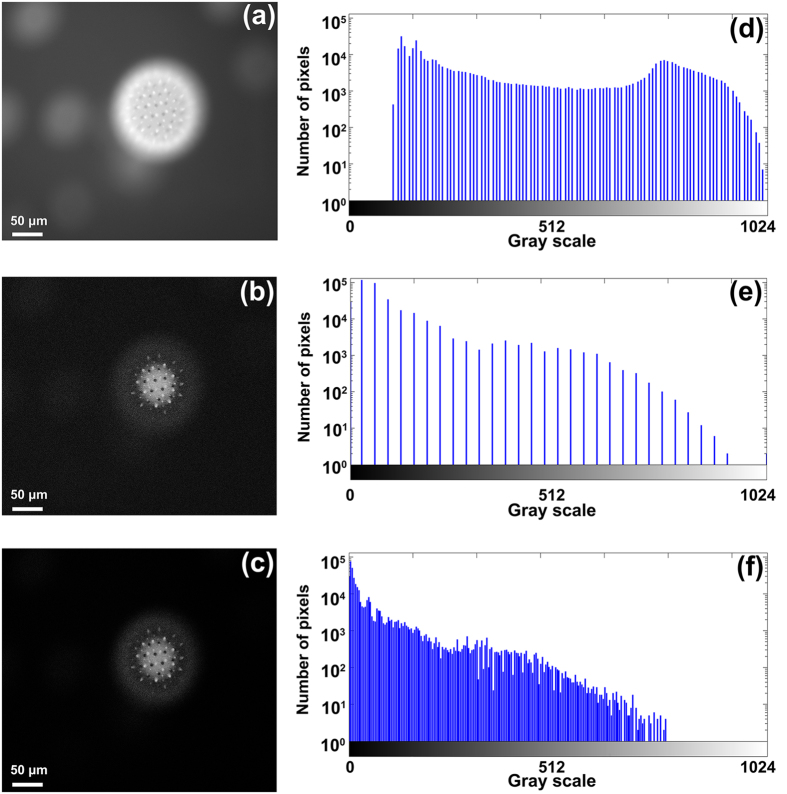
Comparison of wide-field and optical sectioning images of a slice of a pollen grain. (**a**) is the wide-field image. (**b**) is the sectioned image calculated by using Eq. (1). (**c**) is the product of the normalized Fig. 7a,b. (**d**–**f**) give the histograms of Fig. 7a–c, correspondingly.

Because of the subtraction operation in Eq. (1), the above RMS decoding algorithm will lose part of gray scales of the raw image^24^. To illustrate the effect of image dynamic range loss of the RMS decoding algorithm, we first set the CMOS camera working at the monochrome mode and record the auto-fluorescence signal of a pollen grain with our setup. Figure 7a shows the wide-field image of the specimen, Fig. 7b is the optical sectioned image calculated by using the Eq. (1), and Fig. 7c is the product of the normalized Fig. 7a,b. Figure 7d–f give the histograms of Fig. 7a–c, correspondingly. It is seen that Fig. 7a has the full dynamic range of the raw data, but the image appears blurred due to the background fluorescence from the defocus planes. The RMS algorithm of Eq. (1) removes the background but also sacrifices the dynamic range of the raw data. The loss of some gray scales can also be observed from the histograms shown in Fig. 7e. The reduction of gray scales will result in color cast problem in the color restoration. To avoid this problem, we first make a normalization of the intensity of Fig. 7b, and then multiply it with Fig. 7a. By doing so, the reduced dynamic range will be partially restored, as seen in Fig. 7f. With such treatment, the color cast problem for multi-channel integration of color image will be solved in the next step.

### Color restoration in HSV space

The color of images in most electronic display products is formed by combining the trichromatic (Red, Green and Blue) light with varying intensities. Because the R, G and B components of a digital image are not fully independent with each other and the RGB color space does not separate the luminance component from the chrominance ones, the image description in terms of RGB components will suffer from color distortion in the case of processing color images in the RGB channels independently. In contrast to RGB space, HSV color space is much closer to people’s perception of color. This model is based on the principle of color recognized in human vision in terms of hue, lightness and chroma^32^,^33^. HSV is an intuitive model that removes the contact between luminance information and color information, thus, is suitable for color image processing^34^.

**Figure 8:**
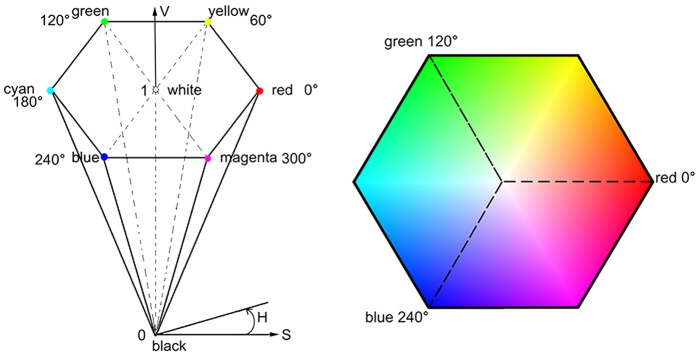
HSV hexahedral cone.

HSV color space is represented with a hexahedral cone as illustrated in Fig. 8. The angle around the central vertical axis corresponds to “hue”, which describes what a pure color is. Starting at the red primary at 0^0^, “H” passes through the green primary at 120^0^ and the blue primary at 240^0^, then wraps back to red at 360^0^. The distance from the vertical axis corresponds to “saturation”, which represents the purity of colors. It takes values from 0 to 1. The height corresponds to the color brightness in relation to the saturation, for which V = 0 means black while V = 1 means white^35^.

**Figure 9:**
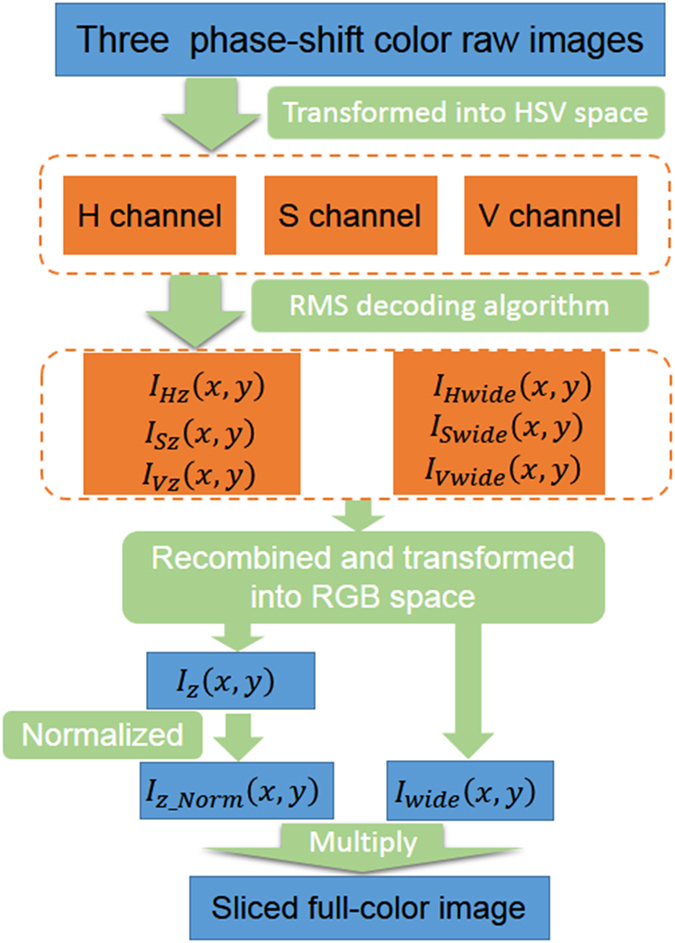
Flowchart diagram for the color restoration procedure of the color SIM.

Figure 9 shows the flowchart diagram of the optical sectioning decoding algorithm for one slice using the proposed full-color structured illumination microscopy. Three raw images with an adjacent phase-shift of 2π/3 are recorded by the color CMOS camera, and transformed from RGB color space into HSV color space to get the three H, S and V components, respectively. Then the RMS decoding algorithm is applied in the three HSV channels separately to obtain the sectioned images of each channel *I*_*iz*_(*x, y*), as well as the wide-field images *I*_*iwide*_(*x, y*), where *i* *=* H, S, V. After that, the sectioned images and the wide-field images for the three HSV channels are recombined and transformed back into the RGB space so as to display the images in devices. It is essential for color restoration to multiply the normalized sectioned image with the wide-field image to recover the loss of some gray scales caused by the RMS decoding algorithm.

## Additional Information

**How to cite this article**: Qian, J. *et al.* Full-color structured illumination optical sectioning microscopy. *Sci. Rep.* **5**, 14513; doi: 10.1038/srep14513 (2015).

## Supplementary Material

Supplementary movie 1

Supplementary movie 2

Supplementary movie 3

Supplementary movie 4

Supplementary movie 5
